# Multiplex Portable Biosensor for Bacteria Detection

**DOI:** 10.3390/bios13110958

**Published:** 2023-10-27

**Authors:** Karim Kaci, Estefanía Enebral-Romero, Emiliano Martínez-Periñán, Marina Garrido, Emilio M. Pérez, David López-Diego, Mónica Luna, Guillermo González-de-Rivera, Tania García-Mendiola, Encarnación Lorenzo

**Affiliations:** 1 HCTLab, Escuela Politécnica Superior, Universidad Autónoma de Madrid, 28049 Madrid, Spain; karim.kaci@estudiante.uam.es (K.K.); guillermo.gdrivera@uam.es (G.G.-d.-R.); 2 Departamento de Química Analítica y Análisis Instrumental, Universidad Autónoma de Madrid, 28049 Madrid, Spain; estefania.enebral@imdea.org (E.E.-R.); emiliano.martinez@uam.es (E.M.-P.); encarnacion.lorenzo@uam.es (E.L.); 3 IMDEA-Nanociencia, Ciudad Universitaria de Cantoblanco, 28049 Madrid, Spain; marina.garrido@imdea.org (M.G.); emilio.perez@imdea.org (E.M.P.); 4 Institute for Advanced Research in Chemical Sciences (IAdChem), Universidad Autónoma de Madrid, 28049 Madrid, Spain; 5 Instituto de Micro y Nanotecnología IMN-CNM, CSIC (CEI UAM+CSIC), 28760 Madrid, Spain; david.lopez.diego@csic.es (D.L.-D.); monica.luna@csic.es (M.L.)

**Keywords:** DNA biosensor, functionalized carbon nanodots, multiplex potentiostat, bacteria detection

## Abstract

An advanced, cost-effective, and portable DNA biosensor capable of detecting multiple bacteria simultaneously has been developed. The biosensor comprises a fast and inexpensive potentiostat that controls the applied potential to a screen-printed electrochemical array platform functionalized with MoS_2_ flakes and bacterial DNA probes. The current response obtained by *à la carte* thionine functionalized carbon nanodots (Ty-CDs) is monitored as an electrochemical indicator of the hybridization event. The design of the potentiostat prioritizes achieving an optimal signal-to-noise ratio and incorporates a user-friendly interface compatible with various devices, including computers, mobile phones, and tablets. The device is compact, lightweight, and manufactured at a low cost. The key components of the potentiostat include a data acquisition board capable of analyzing multiple samples simultaneously and a controller board. The results of this study confirm the ability of the multiplex portable biosensor to successfully detect specific bacterial DNA sequences, demonstrating its reliability and superior performance compared with a traditional, more complex, and laboratory-oriented potentiostat.

## 1. Introduction

Food and water poisoning—a health issue that has affected most of the global population—is caused by myriad sources, including heavy metals, highly toxic pesticides, and foodborne pathogens. Thus, food analysis has become a critical area from a public health and economic point of view. Pathogens can be found in various foods, making their detection imperative to ensure a safe food supply and prevent foodborne diseases. Of all the identified foodborne pathogens in the world, viruses are estimated to be the primary causes of illness, whereas bacteria are the primary causes of hospitalization and death [1]. The common pathogens responsible for most foodborne disease outbreaks include *Listeria monocytogenes* and *Salmonella enterica*, which cause listeriosis and salmonellosis, respectively. These two food- and water-borne diseases have a high infection and mortality rate. The primary symptoms following ingestion are meningitis, septicemia, gastroenteritis, fever, and nausea [2]. Moreover, bacterial detection is especially important in food products and water due to their tendency to spoil or contaminate food, causing serious consumer risks. Currently, polymerase chain reaction (PCR) and enzyme-linked immunosorbent assays (ELISA) are the most common detection methods. Despite the selective, specific, and reproducible detection of target bacteria provided by these techniques, they also require long analysis times and sample pre-treatment and have low sensitivity [3]. Therefore, searching for alternative techniques for early bacterial detection is an area of research interest. Recent studies have focused on evaluating the antibiotic resistance of many bacteria, a major challenge in treating infections. These studies have focused on electro-photonic approaches to analyze the susceptibility of individual bacteria to antimicrobials via changes in their optical properties—changes in wavelength and transmission—and the impedance of the medium surrounding the bacteria. In this way, they discriminate between live and dead bacteria, a major advance in point-of-care diagnostics [4,5,6].

Currently, there is a clear trend toward developing on–off type devices for quick and easy screening of diseases focused on point-of-care in primary care centers or even domestic use. In this sense, electrochemical DNA biosensors have emerged as a potential alternative due to their simplicity, speed, sensitivity, robustness, capacity for miniaturization, automation, and low cost. Although various studies have focused on developing DNA biosensors for bacterial detection [7,8], novel methods and improvements continue to be investigated to achieve multianalyte detection, increased rapidity, specificity, and sensitivity. Indeed, devices capable of detecting different pathogens, including bacteria and viruses, without labelling are under development, primarily focusing on designing different electrodes specific to a particular bacteria [9]. Meanwhile, others have very short analysis times (2–3 min), high sensitivity, and incredibly low detection limits due to the incorporation of different nanomaterials [10,11]. In this sense, research involving nanomaterials in developing new electrochemical biosensors with better analytical properties is particularly interesting. The current study aims to broaden the field by achieving multi-analyte detection of bacteria (*Listeria* and *Salmonella*) by applying nanomaterials. Among the different nanomaterials, molybdenum disulfide (MoS_2_) has become attractive from an electrochemical point of view and in the development of biosensors due to its ability to act as a sensing interface and to interact with thiolated molecules, such as the thiolated DNA probes [12]. The MoS_2_ synthesis method applied in the current study (i.e., liquid phase exfoliation) facilitates the formation of internal and perimeter edges in the crystalline structure of the nanomaterial, which have a high molecular affinity, favoring immobilization of the probe with a specific orientation that could favor subsequent hybridization with the analyte sequence [13].

Due to its simplicity and efficiency, one of the most widely used techniques for detecting the hybridization event in electrochemical DNA biosensors is indirect detection by means of a redox active molecule, known as an electrochemical indicator. These are electroactive substances that have different interactions with double-stranded (ds-DNA) and single-stranded (ssDNA) DNA. In addition, they must exhibit chemical stability of both the oxidized and reduced species, a reversible redox response at low potentials, toxicity, and cost. The signals these molecules provide focus on changes in the potential values or current intensity. Considering the characteristics of a good electrochemical indicator, in this work, we propose using thionine-modified carbon nanodots (Ty-CDs) as a redox indicator. Carbon nanodots (CDs) are a nanocarbon nanomaterial comprising spherically shaped nanoparticles composed of carbon, oxygen, and hydrogen. Their simple bottom-up synthesis methodology facilitates the selection of appropriate precursors to determine their properties and prepare *à la carte* compounds. In this sense, phenazine and phenothiazine compounds have been employed for carbon dot synthesis, granting them excellent optical and electrochemical [14] properties. Despite CDs being employed in DNA hybridization detection strategies [15,16] their use represents an emerging research area to be further exploited. Moreover, the benefit of preparing *à la carte* Ty-CDs with respect to the use of single thionine molecules as electrochemical indicators is the ability to concentrate electrochemical active moieties over the modified electrode, allowing higher electrochemical currents, which improve the transduction sensibility of our devices.

Existing approaches for DNA biosensor-based pathogen detection have limitations regarding multiplex detection, portability, and point-of-care (POC) application. Conventional potentiostats are complex, expensive, and bulky, rendering them unsuitable for POC implementation; budget constraints also pose a significant challenge to developing POC devices based on single-chip potentiostat platforms [17,18]. To address this issue, exploration in the field is ongoing, and one promising technology is the use of single-board computers (SBCs), such as the Raspberry Pi model 3B+ [19]. These SBCs offer high computing power, low power consumption, ease of programming, and cost-effectiveness, making them ideal for tailored solutions in POC and point-of-need applications. The controller module of the electrochemical DNA biosensor system is particularly complex and costly while employing an SBC helps reduce expenses. Furthermore, to equip the device with the capability of analyzing multiple samples simultaneously, a high-frequency multiplexer should be incorporated. Nevertheless, this addition presents the challenge of synchronizing the chemical reaction within the electrochemical cell and configuring the electronic components to deliver the desired signal from the corresponding analyte to the control and signal processing unit to ensure signal and data consistency. To address this issue, a widely adopted engineering resource can be employed: a multiplexation technique. In engineering, this refers to a fundamental technique that optimizes the transmission and reception of multiple signals or data streams over a shared communication channel. The primary objective of multiplexation is to enhance the system’s efficiency by maximizing the utilization of available bandwidth and resources. This technique is particularly valuable in scenarios requiring the transmission of multiple streams of information while avoiding unnecessary duplication of communication channels, which would be impractical and resource-intensive. Hence, in the current study, time division multiplexing (TDM) is employed by allocating discrete time slots to individual signals for sequential transmission.

In this work, we present the development of a multianalyte electrochemical DNA biosensor based on the use of MoS_2_ and *à la carte* thionine functionalized carbon nanodots for the simultaneous detection of *L. monocytogenes* and *S. enterica* coupled with a portable potentiostat specifically designed for micro-power electrochemical sensing applications. The portable potentiostat, built around a Raspberry Pi 3B+ SBC, incorporates filters and analogue-to-digital converters (ADCs) to minimize electrical noise in the system. It also includes a main controller board for fast and accurate sampling that can be operated from any device, including a computer, mobile, or tablet using a WiFi or ethernet connection.

## 2. Materials and Methods

### 2.1. Chemicals

Sodium phosphate monobasic monohydrate (NaH_2_PO_4_·H_2_O), sodium phosphate dibasic (Na_2_HPO_4_), sodium chloride, thionine (3,7-diaminophenothiazin-5-ium chloride), and bulk MoS_2_ and synthetic DNA sequences are summarized in Table 1 and supplied by Merck (https://www.merckgroup.com/ accessed on 4 May 2023). Millipore Milli-Q system 18.2 Ω was used for the experiments.

### 2.2. Apparatus

Electrochemical experiments were performed on an Autolab multi-channel potentiostat from uniscan instrument with UiEChem software, UK. An eight-channel connector (DRP-CAC8X) and eight screen-printed electrode plates (DRP-8X220AT) were used as the interface and transducers, respectively; both were supplied by Metrohm DropSens, Madrid, Spain.

For hardware development, the ALTIUM DESIGNER v21.0.9 PCB design environment was used, as well as the RIGOL DS1104Z oscilloscope, RIGOL DP832 power supply, and JBC NASE-2C soldering station. Regarding the software development, the IDEs PyCharm Version 2023.2.3 and IntelliJ from JetBrains were used.

### 2.3. Procedures

#### 2.3.1. Molybdenum Disulfide (MoS_2_) Flake Synthesis

In a 250-mL round-bottomed flask, 200 mg of the bulk MoS_2_ was dispersed in 200 mL of N-methyl-2-pyrrolidone (NMP) [20], cooled at 2 °C, and sonicated (ultrasonic probe Vibracell 75115, Bioblock Scientific, 500 W, Sonics & materials, Newtown, CT, USA) for 1 h with a 37% amplitude. The resulting black suspension was separated into six 50-mL conical centrifuge tubes and centrifugated for 30 min at 5000 rpm (Allegra X-15R Beckman Coulter centrifuge, FX6100 rotor, 20 °C, Beckman Coulter, Indianapolis, IN, USA). The olive-colored supernatant was separated from the black sediment (non-exfoliated material) by decanting and vacuum filtering (Omnipore 0.45 µm PTFE membrane filters, 45 mm diameter). The retained exfoliated MoS_2_ was cleaned by redispersion and filtration thrice in acetonitrile (60 mL) and thrice in isopropanol (60 mL). The collected solid was dissolved in water and used for further experiments.

#### 2.3.2. Thionine Functionalized Carbon Nanodot (Ty-CD) Synthesis

Ty-CDs were synthesized by irradiating a mixture of 72 mg of thionin acetate salt, 87 mg of L-arginine, 86 µL of 3,3′-diamino-N-methyldipropylamine, and 100 µL of Milli-Q water into a microwave system at 235 °C and 20 bar for 180 s. The blue solid obtained was then dissolved with 10 mL of ultrapure water and filtered with a 0.1 µm porous filter. Then, the suspension was dialyzed for 1 week in a 0.1–0.5 kDa dialysis membrane. Finally, a 2.83 mg/mL Ty-CD solution was obtained and stored at 4 °C.

#### 2.3.3. DNA Solution Preparation

Stock (10 µM) probe solutions were prepared using 10 mM phosphate buffer pH 7.0 solution as the solvent after treatment with dithiothreitol (DTT) and purification through a Sephadex G-25 NAP-10 column. The stock and 100 pM *Listeria* and *Salmonella* target solutions were prepared in 10 mM phosphate buffer pH 7.0 with 0.4 NaCl. All solutions were stored at −20 °C.

#### 2.3.4. Biosensor Platform Development

##### Nanostructuring the Screen-Printed Electrode (SPE) Array

The screen-printed electrochemical array formed by eight electrodes (8xSPE) was nanostructured with MoS_2_ flakes by drop-casting to improve the analytical properties of the biosensor. For this purpose, 5 µL of a colloidal MoS_2_ dispersion in water was deposited on the surface of the gold working electrode by drop-casting over a hot plate to evaporate the organic solvent. The resulting platform was denoted as 8xSPE/MoS_2_.

##### DNA Capture Probe Immobilization

The 8xSPE/MoS_2_ platform was modified with 5 µL of 10 µM thiolated DNA *Listeria* or *Salmonella* capture probes by drop-casting and kept at room temperature for 24 h (SPE/MoS_2_/List-SH or SPE/MoS_2_/Salm-SH). We previously described this methodology using similar platforms, demonstrating that it can be performed at room temperature over 24 h, allowing better DNA probe organization [12,16]. The platform was then washed with Milli-Q water.

##### Hybridization Reaction and Electrochemical Detection

The platform with the immobilized probe was hybridized with 5 µL of 100 pM *Listeria* or *Salmonella* target sequences. Optimal conditions were obtained for 1 h at 40 °C using a humidity chamber, as previously reported for similar platforms [21]). To detect the hybridization event, carbon nanodots modified with thionine (Ty-CDs) were used as redox indicators. For this purpose, 5 µL of 2.83 mg/mL Ty-CDs solution was incubated for 1 h at room temperature over the working electrode [16]. The electrodes were rinsed with Milli-Q water, and the differential pulse voltammograms (DPV) were recorded in each case using PB 0.1 M pH 7.0 as the supporting electrolyte. Measurements were performed on the designed portable multiplex potentiostat (single-pulse potential measurement) and the commercial Autolab multiplex potentiostat for comparison.

##### Biosensor Selectivity Analysis

To study the selectivity of the biosensor, 8xSPE/MoS_2_/List-SH and 8xSPE/MoS_2_/Salm-SH platforms were incubated under the same conditions (1 h, 40 °C) with *Salmonella* and *Listeria* target sequences acting as non-complementary sequences. In the same way, they were incubated for 1 h with Ty-CDs, the electrodes were washed with sterile water, and the DPVs were recorded in 0.1 M PB pH 7.0.

#### 2.3.5. Multiplex Potentiostat Design and Development

The developed multiplex potentiostat presents a comprehensive device for conducting end-to-end measurements meticulously executed at the hardware and software levels.

**Scheme 1:**
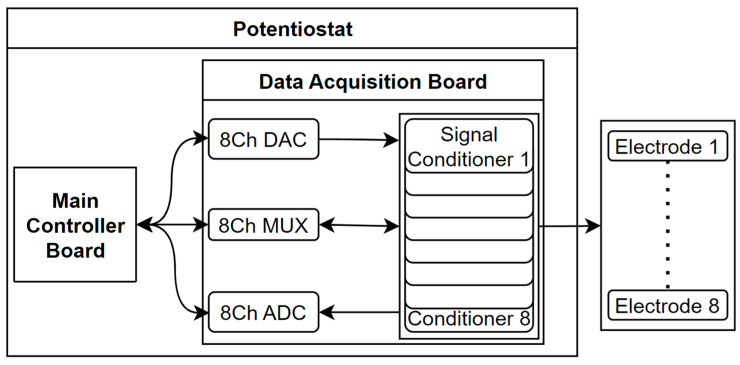
Hardware design scheme.

At the hardware level, the design led to the creation of two distinct modules: the main controller board and the data acquisition board. The former acts as a repository for all necessary software and oversees the system’s functioning, running all necessary algorithms and commanding the second module, comprising three modules: the data multiplexing, signal conversion, and signal acquisition modules. The first module represents the core, permitting multiple measurements by routing the signals between all chemical cells and the main controller module. The second module incorporates the essential electronic components required to process the signal, including ADCs and DACs and a set of filters that achieves a low electrical noise level in the system. The third module comprises all essential electronic components and signal conditioners necessary to acquire the raw signal from the electrochemical cell. A scheme of the hardware design is presented in Scheme 1.

**Scheme 2:**
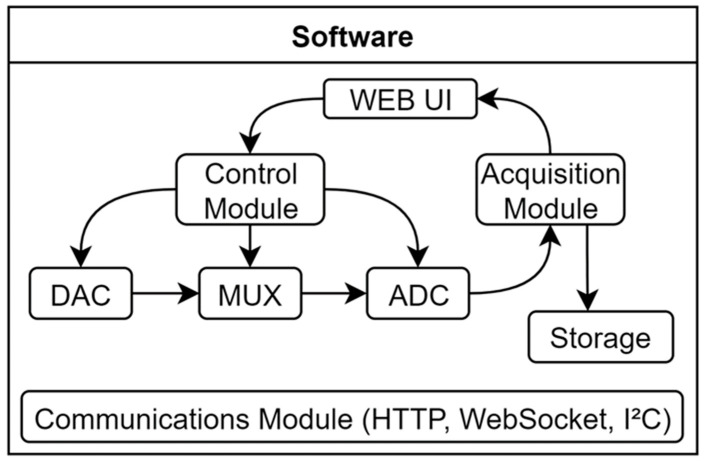
Software design scheme.

The software-level design yielded five modules: the communications module, which is responsible for managing the data transfer among all system components; the control module, which governs the behavior of each individual component within the system; the digital filter module digitally processes the acquired signal using the Savitzky-Golay algorithm; the data acquisition module obtains and processes the signal from the biosensor; the user web interface module serves as the user interface for the operator, allowing for seamless interactions with the multiplex potentiostat system. A scheme of the software design is presented in Scheme 2.

##### Main Controller Board

The primary objective of the main controller board is to provide an affordable and portable device. To achieve this, the design emphasizes modularity, aiming to utilize commercially available modules as much as possible. This approach reduces the need for custom electronic boards, thus lowering manufacturing costs. Among the various modules within the system, the controller module is particularly complex and expensive. In the prototyping phase, a complete computer was initially used. However, to reduce costs, a commercial development board was chosen as the controller.

Several development boards, including *Arduino YUN* (Mouser, Barcelona, Spain), *BeagleBone Black*, and *Raspberry Pi model 3B+* [22,23,24], were evaluated to determine the most suitable option for the project. Considerations such as communication and processing capabilities, as well as flexibility in software development and maintenance, were considered during the selection process. Based on the analysis, the *Raspberry Pi 3B+* development board was deemed the most suitable due to its high core speed, communication capabilities, and cost-effectiveness. Although it lacks analogue ports, this can be easily resolved by incorporating an ADC.

Another advantage of the selected development board is that it features HDMI and USB ports, enabling standalone usage by simply connecting a screen, mouse, and keyboard. However, to utilize the *Raspberry Pi 3B+* board, additional elements are required, including a μSD card with a minimum capacity of 4 GB and a 5 V μUSB power adapter with a current output of 2.5 A. Additionally, an ethernet cable can be used to connect to the board through the SSH protocol, or alternatively, an HDMI screen with a USB mouse and keyboard can be employed.

##### Data Acquisition Board

The data acquisition board serves as an integration point for essential electronic components involved in the sensing and physical treatment of the signal generated during the chemical reaction. These components are configured and controlled by the controller board, and the captured signal is subsequently returned to it for further processing. The primary modules housed on the data acquisition board include the data multiplexing module, which enables the routing of the signal from the electrochemical cell to the main controller board using the TDM technique; the signal acquisition module, which comprises several signal conditioners; the signal conversion module, including the ADCs and DACs; and a set of non-configurable components that helps maintain a low noise level within the system.

To facilitate the required functionalities and interactions with the controller board, the data acquisition board incorporates the I^2^C communication bus [25] through the SCL (serial clock) and SDA (serial data) ports. This communication enables the programming of the components and the retrieval of measurement data from the sensor.

The data acquisition board is designed so that it is connected to the main controller board through its 40-pin connector. This allows the two modules to be physically abstracted so that if one of the modules breaks, only that element has to be replaced, not the entire system. Furthermore, since the data acquisition module is designed with two layers of the printed circuit, if any of its components fail, the piece in question can be desoldered and replaced, facilitating an easy repair process. Meanwhile, due to the complexity and low cost of the control module, in case of failure, it is recommended to replace, rather than repair, the module. Additionally, the data acquisition board can be connected to any future version of the SBC so long as the same connection design is retained.

The multiplexing module is the core circuit that facilitates multiple measurements using the same device and is based on a key component: the *Texas Instruments PCA9548 8-channel I^2^C-bus multiplexer* (Mouser, Barcelona, Spain). This chip plays a crucial role as it provides up to eight channel communications, enabling up to eight electrochemical cells to perform up to eight measurements on distinct analytes during the same execution. This facilitates the analysis of multiple samples simultaneously, understanding the simultaneity not as parallel execution but as obtaining all results at the end of an operational window, with a maximum capacity of eight samples. The multiplexer uses a TDM technique by selectively exposing one of the connected signal conditioners, connected to a chemical cell, and routing the I^2^C bus signals of the desired channel to the controller board. This functionality allows for the parallel processing and analysis of multiple samples, providing greater efficiency and throughput. By leveraging the capabilities of the PCA9548 multiplexer, the system gains the flexibility to handle more samples and perform several measurements, enhancing the overall performance and productivity of the electrochemical sensing setup. Moreover, the signal acquisition module, LMP91000, has a non-configurable I^2^C address; hence, without a multiplexing technique, the controller board could work only with one electrochemical cell.

The signal acquisition module is based on the *Texas Instruments LMP91000 configurable AFE Potentiostat* (Mouser, Barcelona, Spain), specially designed for low-power chemical sensing applications, as the signal conditioner. This component is critical in conditioning and preparing the raw signal for further processing. It may involve amplification, filtering, and any necessary adjustments to ensure the signal is suitable for subsequent conversion and analysis. Furthermore, the signal conditioner requires a reference voltage supplied through the *V_ref_* port. This reference voltage is essential for performing a potential sweep on the chemical sample, enabling precise control and measurement of the electrochemical reaction. To ensure the integrity and quality of the acquired signal, the conditioner incorporates a capacitor connected to ports *C1* and *C2*. This capacitor is vital to analogue filtering, effectively attenuating electrical noise and disturbances from the signal. The result is an output signal, available at *V_out_* port, that is as clean and noise-free as possible, enhancing the accuracy and reliability of the measurements.

The *Raspberry Pi 3B+* development board lacks analogue GPIOs and can, therefore, only handle digital signals. To overcome this limitation, the signal conversion module was designed using ADCs and DACs. The *Texas Instruments ADS1115* (Mouser, Barcelona, Spain) is responsible for converting the analogue signal received from the biosensor—the *V_out_* port of the signal conditioner—into a digital format that the controller board can process. This component boasts 16-bit resolution, which translates to greater precision in working with the signals, and the sigma–delta conversion procedure, which offers several advantages, including inherent linearity, monotonicity, and minimal requirements for external anti-aliasing filters. The digital nature of this conversion method ensures that the performance remains stable over time and temperature. However, it requires complex digital circuitry and operates at an oversampled rate, significantly higher than the maximum signal bandwidth. Conversely, the *Microchip MCP4728* (Mouser, Barcelona, Spain), is utilized to convert digital signals from the controller board into analog signals, *V_ref_* signal, used by the signal conditioners. These integrated circuits are specifically designed to operate with high precision and low noise, crucial factors for handling weak signals and minimizing electrical noise susceptibility. This component incorporates a high-precision output amplifier, enabling precise and stable output voltage generation.

Additionally, the data acquisition board incorporates various non-configurable components that help maintain a low noise level in the overall system. These components work in tandem to reduce or eliminate unwanted electrical noise, interference, or disturbances that can adversely affect the quality and accuracy of the acquired signal. Capacitors are involved in the power line to ensure a smooth clean power signal throughout the circuit. Additionally, decoupling capacitors are placed at the power entry of each integrated circuit on the board to clean the power signal for each chip. The *Texas Instruments OPA388 Precision Operational Amplifiers* (OPAs) (Mouser, Barcelona, Spain) are strategically placed in buffer configuration between the signal conditioners and ADC/DAC integrated circuits. This component is designed to meet high precision and ultra-low noise requirements and integrates filters to mitigate electromagnetic and radiofrequency interference.

Together, these components serve as a vital intermediary between the physical sensing of the chemical reaction and digital processing performed by the controller board. They enable efficient signal capture, conditioning, and conversion, ensuring that the acquired signal is of sufficient quality and fidelity for subsequent analysis and interpretation by the system.

##### Software Design

The software implementation of the device can be divided primarily into the back-end and front-end. The back-end comprises the software necessary for managing the system but is not directly accessed by the operator. This includes the communication protocols between the various components of the device, internal controls of each chip, digital filtering algorithms, and data acquisition algorithms. Thus, the back-end ensures the commands are correctly interpreted and executed. Meanwhile, the front-end encompasses the software that allows the operator to interact with and manage the system. This includes a user web-based interface where the operator can visualize the results and control various aspects of the POC device. The front-end module is designed to be user-friendly and accessible, enabling operators to work from any device, such as a computer, tablet, or mobile phone.

Together, these software modules contribute to the efficient functioning and usability of the system, enabling seamless communication, precise signal treatment, accurate data acquisition, and an intuitive user experience.

##### Communications Module

The communications module plays a crucial role in facilitating communication between different components of the POC device and enabling interaction with the operator through the user interface. This module utilizes various protocols to ensure effective and reliable data transfer.

The I^2^C protocol is utilized to communicate between the controller board and the data acquisition. It enables the controller board to configure and control the multiplexer, signal conditioner, ADC, and DAC on the data acquisition board. Through I^2^C, the controller board can send commands and receive data from these components, facilitating the overall operation of the POC device.

The TCP (transmission control protocol) is employed for data transfer between the operator’s user interface (UI) and device, whereas the HTTP (hypertext transfer protocol) is specifically used within the TCP to enable the operator to interact with the POC device through a web-based UI. The HTTP protocol allows the operator to send commands, configure device settings, and retrieve information from the device in a user-friendly manner.

In addition to HTTP, the communications module utilizes WebSocket, which is built on top of the TCP, to achieve real-time data display on the UI plot. WebSocket enables a persistent, full-duplex communication channel between the UI and device. This allows the acquired data to be streamed and displayed on the UI plot in real time, providing the operator with immediate feedback and visualization of the measurements.

By leveraging these protocols, the communications module ensures seamless communication between the different components of the POC device and facilitates user interaction through a web-based UI. This enhances the usability and functionality of the device, enabling efficient configuration, control, and real-time data visualization for the operator.

##### Control Module

The control module of the software is responsible for handling the commands received from the operator through the web-based UI and configuring the internal registers of the data acquisition board components. An overview of the configuration requirements is presented here:

1. Multiplexer (*PCA9548*): The control module configures the multiplexer to select the desired channel corresponding to a specific signal conditioner on the data acquisition board. This allows the system to select which signal conditioner to expose for measurement, allowing the analysis of up to 8 samples simultaneously; simultaneously refers to obtaining all results at the end of the same operational window.
2. Signal conditioner (*LMP91000*): The control module configures internal registers of the signal conditioner based on the operator’s commands. This configuration includes:
  2.1 Transimpedance control register (*TIACN*): Sets the amplifier gain (*RTIA*) and load resistance (*RLOAD*).
  2.2 Reference control register (*REFCN*): Determines the voltage reference (*V_ref_*) source (internal or external), sets the internal zero value as a percentage of *V_ref_*, and configures the bias sign and value as a percentage of *V_ref_*.
  2.3 Mode control register (*MODECN*): Enables or disables the *FET* feature and selects the operation mode of the sensor.

The control module ensures that the signal conditioner is properly configured to provide the desired amplification, reference voltage, biasing, and operating mode for the electrochemical cell.

3. DAC (*digital-to-analogue converter*): The DAC integrated circuit on the data acquisition board has a single register that the control module must configure. This register sets the desired output voltage, which will be used as the *V_ref_* (reference voltage) for the signal conditioner. A variable voltage reference makes the device very flexible, as it can be configured to analyze a large diversity of samples.
4. ADC (analogue-to-digital converter): The ADC integrated circuit on the data acquisition board also requires configuration, however, these settings are handled automatically and are not accessible for operator modification. The control module ensures that the ADC is properly calibrated and set up during system start-up, allowing it to convert the analogue signal obtained from the signal conditioner accurately.

By properly configuring the registers of each component, the control module ensures that the data acquisition board is correctly set up for signal conditioning, conversion, and measurement of the electrochemical cell’s output.

##### Digital Filter Module

The digital filter module in the software implementation is embedded in the data acquisition module and utilizes a *Savitzky-Golay* [26,27,28,29,30] filter to enhance the precision of the data obtained from the ADC. The *Savitzky-Golay* filter is a type of digital *finite impulse response* (FIR) filter that aims to improve the accuracy of the data without significantly distorting the signal trend.

The filter operates through convolution, where successive subsets of adjacent data points are fitted with a low-degree polynomial using the method of linear least squares. By finding a set of “convolution coefficients,” which can be determined analytically when the data points are equally spaced, the filter estimates the smoothed signal at the central point of each subset.

The original work by Savitzky and Golay demonstrated that the smoothed output value obtained by sampling the fitted polynomial is equivalent to a fixed linear combination of the local set of input samples. Hence, the output samples can be computed through discrete convolution.

The *Savitzky-Golay* filter has two key parameters: the window length, which determines the number of samples used for the filtering process, and the polynomial order, which specifies the fitting polynomial degree. These parameters are calibrated and set up in the control module of the software, ensuring optimal filtering performance. However, they are not accessible for modification by the operator. Nevertheless, by applying the *Savitzky-Golay* filter, the digital filter module enhances the representation of the signal, providing a smoother and more precise visualization of the data for the operator.

##### Data Acquisition Module

The data acquisition module is responsible for sampling the *V_out_* output signal from the signal conditioner and processing it for various purposes. First, the module is responsible for presenting the acquired data on the UI, allowing the operator to monitor the measurements and observe any changes or trends in the data. Additionally, the data acquisition module facilitates data storage for future reference and analysis. The acquired data is saved in a CSV (comma-separated values) file, a common format easily accessed and processed by various third-party applications, such as spreadsheet software or data analysis tools. Storing the data in a CSV file enables the operator to retrieve and utilize it outside the POC device, enhancing its usability and compatibility with other applications or analysis workflows.

##### User Web Interface Module

The user web interface module provides a user-friendly and intuitive control panel to the operator, enabling the configuration of various system parameters with ease. The interface offers a simple and interactive interface where the operator can make selections to adjust settings or choose from pre-set configurations. The user web interface module also facilitates the visualization of the analysis results in graphical form, allowing the operator to readily interpret and analyze the data obtained from the biosensor. The graph provides a visual representation of the measurements, trends, and changes over time, aiding in the understanding of the analyzed samples.

A key advantage of the user web interface module is its accessibility. Being a web-based interface, it can be accessed from any device connected to the same local network as the POC device. This eliminates the dependency on a specific computer or the need for installing third-party drivers. The operator can access and control the system from their computer, laptop, tablet, or mobile phone, providing flexibility and ease of use. Hence, the user web interface module enhances the overall user experience by offering a user-friendly interface, real-time visualization of results, and accessibility from various devices within the local network. In this way, it simplifies the control and monitoring of the system, making it more convenient and efficient for the operator.

## 3. Results

**Scheme 3:**
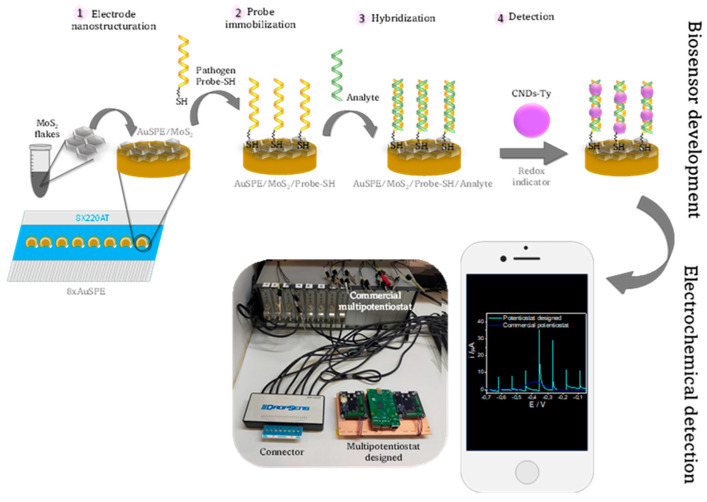
Scheme for the multiplex portable DNA sensing device development.

In this work, we propose the development of a multiplex portable device capable of easily and rapidly detecting *L. monocytogenes* and *S. enterica* with high sensitivity at the point of care. Hence, this study has focused on the design and development of a portable, simple, and rapid multiplex potentiostat coupled to an electrochemical DNA sensing platform. Scheme 3 presents the main components (electrochemical DNA biosensor and portable multiplex potentiostat) and the principal steps for the multiplex portable device development.

To develop a portable multiplex potentiostat capable of handling multiple analytes, the hardware and software must be effectively designed. This portable concept of the device requires a miniaturization of the electronics. Meanwhile, the primary obstacle in this work was providing the system with the ability to process the signal from several electrochemical cells simultaneously. To achieve this, time division multiplexing [31] was applied, obtaining a device capable of analyzing up to eight samples at the same time.

The electrochemical DNA biosensor preparation (Scheme 3) is based on the use of two different nanomaterials, MoS_2_ for nanostructuring the SPE surface and *à la carte* Ty-CDs for hybridization detection. As shown in Scheme 3, the first step in the biosensor development is creating a nanostructure with MoS_2_ of screen-printed electrodes (SPE). This nanomaterial improves the analytical properties of the biosensor, generating a stable platform (MoS_2_/SPE) for the immobilization of the specific bacterial DNA probe [16]. The second stage is the immobilization of the thiolated DNA probes (List-SH or Salm-SH) on the SPE/MoS_2._ This is achieved by the ability of the thiol group to passivate the vacancies in the MoS_2_ layer on the electrode surface, leaving the probe available for subsequent hybridization with its complementary sequence [12]. The next step is the hybridization of the platform with the analyte (i.e., complementary (List-C or Salm-C) or non-complementary sequences). Finally, the detection of the hybridization event was carried out using Ty-CDs as redox indicators. Indeed, these nanomaterials are efficient redox indicators [16]. Further Ty-CD characteristics are described in the Supporting Information (see Figure S1). To select the optimal procedure for incorporating the redox indicator into the dsDNA immobilized layer, two different strategies were investigated. That is, the complementary sequences were incubated with Ty-CDs incorporated by cycling the potential from −0.70 to −0.15 V or via accumulation at open potential; the resulting biosensor signals in 0.1 M PB pH 7.0 buffer are depicted in Figure S2. Both strategies incorporated the redox indicator in the immobilized DNA layer, facilitating the accumulation of the redox indicator. However, the peak current ascribed to the oxidation of the Ty-CDs by incubating at open potential was higher than that obtained by successive scans of potentials suggesting that more Ty-CDs were accumulated. Considering these results, the accumulation of the redox indicator via incubation at open potential was selected as the most efficient strategy for POC system development.

**Figure 1:**
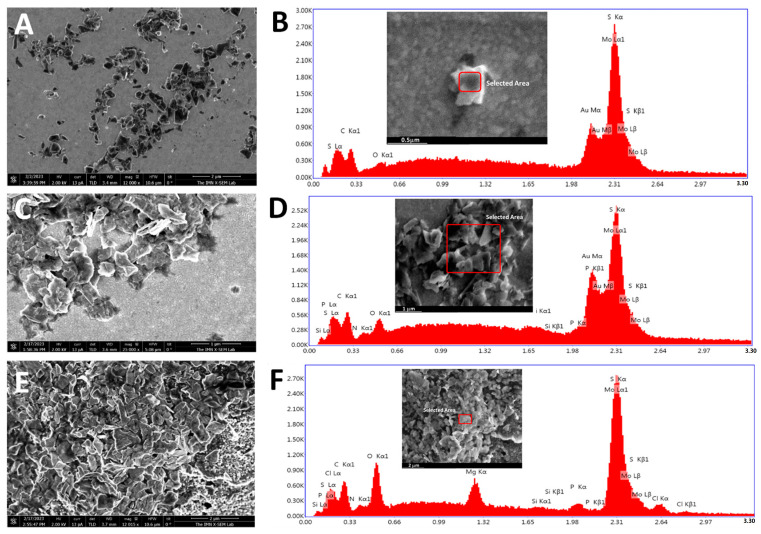
SEM (**A**,**C**,**E**) and EDAX (**B**,**D**,**F**) of a MoS_2_-modified gold substrate; (**A**,**B**) probe immobilization on the MoS_2_-modified gold substrate; (**C**,**D**) probe immobilization on the MoS_2_-modified carbon substrate. (**E**,**F**) The images show the triangular structures of MoS_2_ flakes on the substrates. The presence of DNA can be identified by the presence of P in the EDAX spectra. The peaks with the energies corresponding to P are more readily distinguishable when the substrate is not gold (**F**) than when it is gold (**D**), as the EDAX energy peaks for Au and P are similar.

The biosensing platform was characterized by SEM-EDAX, AFM, and fluorescence microscopy. Figure S3 shows the AFM (Figure S3A,B) and SEM (Figure S3C) images and an EDAX (D) spectra of a bare gold substrate (i.e., a surface composed of Au nanocrystals). After MoS_2_ modification (Figure 1A,B), a homogeneous distribution of the MoS_2_ onto the Au surface was observed (Figure 1A). Moreover, from the EDAX spectrum, the peaks corresponding to the energies of Mo, S, and Au were identified, confirming the MoS_2_ modification. After probe immobilization (Figure 1C–F), the peak corresponding to the P and N energies from the DNA probe was detected. Since the energy peaks of P and Au were similar, they overlapped on the probe immobilization gold platform spectrum (Figure 1D). To avoid this, the gold substrate was substituted with carbon (Figure 1E,F) to ensure that the P peak was readily differentiated (Figure 1F), confirming the presence of DNA on the MoS2-modified platform. The other peaks correspond to the MoS_2_ solvent and the buffer solution used for the DNA probes.

**Figure 2:**
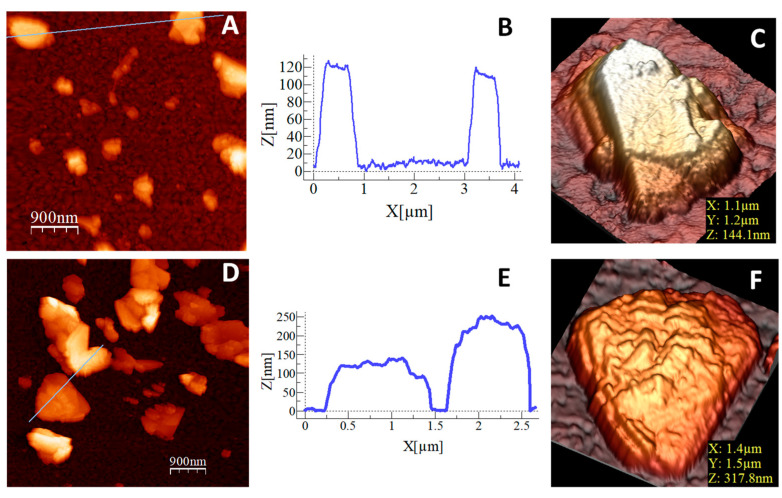
AFM images of (**A**) Au/MoS_2_ and (**D**) Au/MoS_2_/Probe_FAM_-SH, their respective profiles (**B**,**E**), and three-dimensional AFM images (**C**,**F**).

Figure 2 presents the AFM images [32], as well as the profile and 3D representation of the MoS_2_ topography on gold (Figure 2A–C) and MoS_2_/Probe_FAM_-SH on gold (Figure 2D–F). Flakes composed of different numbers of stacked layers can be observed. The gold topography image and profile are shown in Figure S3.

**Figure 3:**
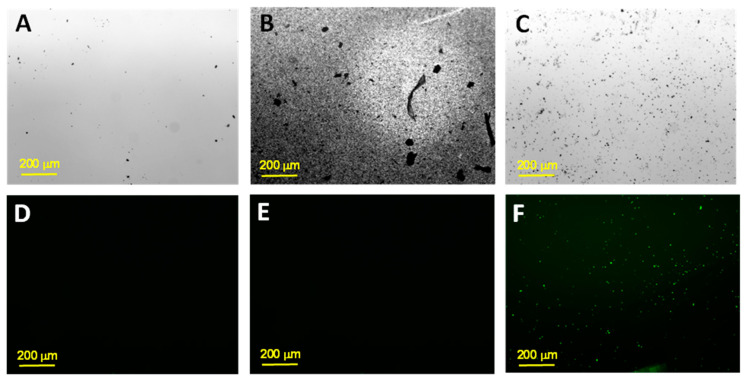
Bright-field (**A**–**C**) and fluorescence (**D**–**F**) optical images of the bare gold substrate (**A**,**D**), Au/MoS_2_ (**B**,**E**) and Au/MoS_2_/Probe_FAM_-SH (**C**,**F**). Only the Au/MoS_2_/Probe_FAM_-SH shows contrast on the fluorescence image (**F**): bright dots that correspond to the black dots (MoS_2_ flake aggregations) of image (**C**).

Fluorescence microscopy images were also captured to follow the probe immobilization onto Au/MoS_2_, a thiolated probe modified with a fluorophore (FAM) was employed. The bright-field optical microscopy images before (Figure 3B) and after (Figure 3C) probe immobilization are similar. However, the fluorescence images (Figure 3D–F) differed as only the Au/MoS_2_/Probe_FAM_-SH (Figure 3F) produced a fluorescence image with contrast, confirming that the DNA probe was bound to Au/MoS_2_ (Figure 3F). Meanwhile, neither bare Au (Figure 3D), nor Au modified with Au/MoS_2_ (Figure 3E) exhibited fluorescence contrast.

**Figure 4:**
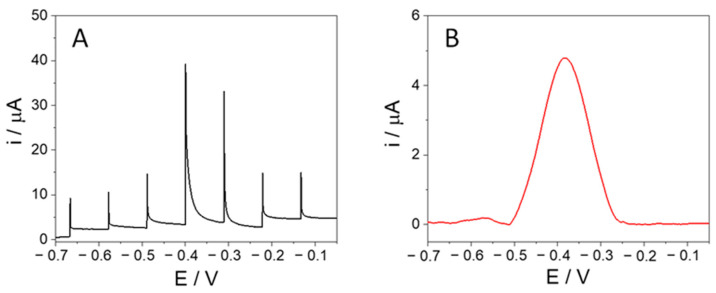
Signal registered for the platform SPE/MoS_2_/Salm-SH after incubating with 10 µL of 100 pM *Salmonella* complementary sequence with the designed portable multiplex potentiostat (**A**) and the commercial multiplex potentiostat (**B**).

Once we confirmed DNA biosensor development, we validated the usefulness of the designed multiplex potentiostat. To this end, we compared the biosensor response obtained by the commercial potentiostat to that of the designed portable multiplex potentiostat before and after hybridization with the complementary sequence. Figure 4 shows the intensity registered after applying different potentials with the portable multiplex (Figure 4A) and the commercial (Figure 4B) potentiostats. The maximum current was recorded, in both cases, at a potential of approximately −0.4 V, ascribed to the oxidation of the redox indicator (Ty-CDs). Meanwhile, the intensity at this potential was higher in the case of the portable multiplex potentiostat (40 µA) than the commercial potentiostat (5 µA). Hence, besides being more accessible and portable, the designed multiplex potentiostat is more sensitive than the commercial system. This is another advantage when applying the potentiostats as POC devices. 

Regarding the shapes of the curves, in the case of the portable multiplex potentiostat, continuous sampling of the current in the cell was carried out; the graph represents the current against the established potential (not against time). Since the potentiostat maintains the voltage constant, the baseline current tends to be 0 µA, whereas when a change in potential occurs, corresponding to the voltage variation, a current peak is detected, tending to drop to 0 µA once the potentiostat has stabilized the desired voltage. Hence, a base current close to 0 µA is visible with peaks at the voltage changes.

**Figure 5:**
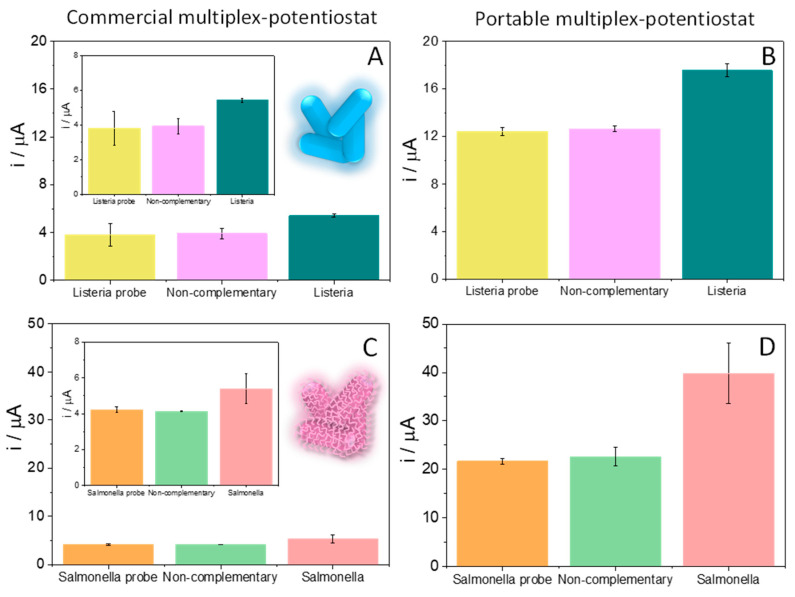
Maximum current intensity obtained at a voltage of −0.4 V with the commercial multiplex potentiostat (**A**,**C**) and portable multiplex potentiostat (**B**,**D**) for the SPE/MoS_2_/List-SH (lime), SPE/MoS_2_/List-SH/NC (purple), SPE/MoS_2_/List-SH/List-C (blue), CSPE/MoS_2_/Salm-SH (orange), SPE/MoS_2_/Salm-SH/NC (green), and SPE/MoS_2_/Salm-SH/Salm-C (pink) platforms.

To confirm multianalyte detection, we evaluated the biosensor response (at −0.4 V) using both potentiostats before and after hybridization with the specific sequence of each bacterium (List-C or Salm-C) and with a non-complementary sequence. For both analytes, a clear difference can be observed in the intensity of the current recorded when the platform was hybridized with the complementary sequence compared with the unhybridized control (Figure 5). In contrast, when the platform was hybridized with a non-complementary sequence, the current intensity was similar to that of the control. These results confirm the applicability of the developed device to multiplex detection of specific bacterial sequences of interest using Ty-CDs as electrochemical indicators and MoS_2_ as an immobilization platform. Furthermore, the signals obtained using the portable multiplex potentiostat were at least 3 times higher than those obtained with the commercial potentiostat, suggesting a better signal/noise ratio in the developed potentiostat.

The reproducibility and repeatability of the test were evaluated based on the signals obtained for three independent measurements of three replicates for the device following the procedure discussed in the Materials and Methods section for the development of the sensing platform. Values of the maximum current intensity recorded at −0.4 V obtained with their error bars shown in Figure 5, are the mean of the three measurements and their standard deviations, respectively. From these results, it can be concluded that the experimental limit of detection obtained for both analytes was 100 pM.

**Figure 6:**
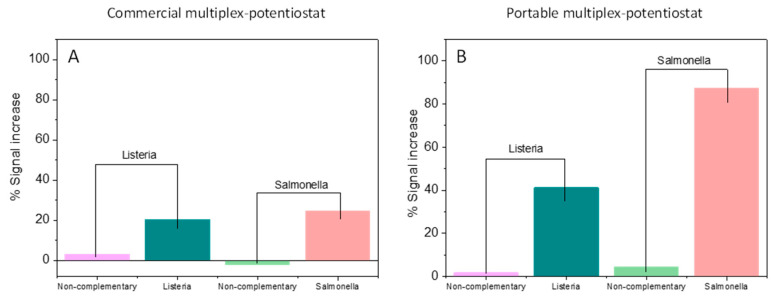
Comparison of the % signal increase obtained with the commercial multiplex potentiostat (**A**) and portable multiplex potentiostat (**B**) after incubating the SPE/MoS_2_/List-SH and the SPE/MoS_2_/Salm-SH platforms with a non-complementary sequence (purple and green), or *Listeria* and *Salmonella* complementary sequences, respectively (blue and pink).

To obtain a better comparison of the results obtained using both potentiostats, we represented the percentage of the signal increase after incubating the SPE/MoS_2_/List-SH and SPE/MoS_2_/Salm-SH platforms with non-complementary and complementary sequences using the commercial (Figure 6A) and developed multiplex potentiostat (Figure 6B). The percentage of the signal increase was calculated following the expression: % signal increase = [(Analyte signal after hybridization − Blank signal/Blank signal)]·100, where the blank signal is the signal obtained for the platforms SPE/MoS_2_/List-SH and SPE/MoS_2_/Salm-SH and the analyte signal is that obtained after incubation with a non-complementary sequence or the *Listeria* and *Salmonella* complementary sequences, respectively. The % signal increase registered with the portable multiplex potentiostat after the hybridization event was higher than that obtained with the commercial system (three times higher in *Salmonella* detection; Figure 6). Thus, confirming the advantages in sensitivity of the designed biosensor in terms of multiplex bacteria biorecognition.

## 4. Conclusions

In this study, a portable DNA biosensor for the multiplex detection of bacteria (*Listeria* and *Salmonella)* is presented, serving as a proof-of-concept for a POC DNA sensing device. The biosensor combines a disposable DNA sensing electrochemical platform based on the use of MoS_2_ and *à la carte* Ty-CDs with a portable multiplex potentiostat for signal acquisition. The design, construction, and validation of each component of the prototype are described in detail. The system is constructed with a main controller board responsible for overall equipment management, and a data acquisition board housing the necessary components for fast and low-noise data acquisition.

The primary objectives of this work were to develop a portable, cost-effective, and user-friendly DNA biosensing device for the specific detection of *Listeria* and *Salmonella* DNA sequences. These objectives were achieved through compact dimensions, affordability, and the ability to interface with a computer, smartphone, or tablet via a local network connection or the internet. The device offers a user-friendly interface for operation and control.

The portable multiplex potentiostat was validated by successfully detecting the specific DNA sequences of two bacteria. The performance of the device was comparable to that of a commercial potentiostat, indicating the advantages in sensibility and potential application of the prototype in POC testing scenarios. Overall, this work presents a promising development in the field of multiplex DNA biosensing.

## Figures and Tables

**Table 1:** Synthetic DNA sequences used in this work.

Synthetic DNA Sequences		
Listeria probe	5′-[SH-C_6_ H_12_]-CCTAGCAGGTCTAACCGCACTCACT	List-SH
Salmonella probe	5′-[SH-C_6_ H_12_]-GCCGCGCGCGAACGGCGAAGCGTAC	Salm-SH
Listeria target	AGTGAGTGCGGTTAGACCTGCTAGG	List-C
Salmonella target	GTACGCTTCGCCGTTCGCGCGCGGC	Salm-C
Probe-FAM	5′-[SH-C_6_H_12_]-CCATAACCTTTCCATTTTTTTTTTACATTCCTAAGTCTGAAACATTACAGCTTGCTACACGAGAAGAGCCGCCATAGTA3’-[6-FAM]	SH-Probe-FAM

## Data Availability

Not applicable.

## Supplementary Materials

The following supporting information can be downloaded at: https://www.mdpi.com/article/10.3390/bios13110958/s1. Figure S1. Ty-CDs characterization. Figure S2. DPV of the biosensor signal after accumulating Ty-CDs by different methodologies. Figure S3. AFM image, SEM image and EDAX spectra of a bare gold substrate.
