# Supplementary material for: Multiplex Portable Biosensor for Bacteria Detection
Source: Biosensors (Basel). 2023 Oct 27;13(11):958. doi: 10.3390/bios13110958 (PMC10669278; doi:10.3390/bios13110958)
Supplement: Supplementary file 1 [file biosensors-13-00958-s001.zip › biosensors-2632591-supplementary.pdf]

Supporting Information

Multiplex Portable Biosensor for Bacteria Detection

Karim Kaci [1,†], Estefanía Enebral-Romero [2,3,†], Emiliano Martínez-Periñán [2,4], Marina Garrido [3], Emilio M. Pérez [3], David López-Diego [5], Mónica Luna [5], Guillermo González-de-Rivera [1], Tania García-Mendiola [2,4,*] and Encarnación Lorenzo [2,3,4]

[1] HCTLab, Escuela Politécnica Superior, Universidad Autónoma de Madrid, 28049 Madrid, Spain
[2] Departamento de Química Analítica y Análisis Instrumental, Universidad Autónoma de Madrid, 28049 Madrid, Spain
[3] IMDEA-Nanociencia, Ciudad Universitaria de Cantoblanco, 28049 Madrid, Spain
[4] Institute for Advanced Research in Chemical Sciences (IAdChem), Universidad Autónoma de Madrid, 28049 Madrid, Spain
[5] Instituto de Micro y Nanotecnología IMN-CNM, CSIC (CEI UAM+CSIC), 28760 Madrid, Spain
* Correspondence: tania.garcia@uam.es
† These authors contributed equally to this work.

Results and Discussion

TEM micrograph (Figure S1A) shows Ty-CDs with quasi-spherical shape. In the inset of Figure 1A (histogram) we have estimated by measuring the average size of around 120 Ty-CNDs and the result indicates a mean size of 3.1 nm ranging from 1.5 to 5.0 nm in diameter. Figure S1B shows the FT-IR spectrum of Ty-CDs, CDs and thionin acetate spectra. The FT-IR spectrum of Ty-CDs is quite similar to that of CDs; however, the increase of the band at 1617 cm^{-1} and the wide band at 3436 cm^{-1} are consistent with the presence of a great number of amine groups due to the thionin presence, suggesting thionin insertion into CDs structure. Although these results are not conclusive, they indicate small compositional changes that agree well with the presence of thionin covalent linked with carbon dot structure. The UV-Vis spectrum (Figure S1C) of Ty-CDs (black) shows an absorption peak at 285 nm, related with the contribution of π-π^* transition of the conjugated C=C units due to carbon dots nanostructure, as it is observed in CDs spectrum (blue). The peak ascribed to the thionin covalently linked with the CDs is centered at 603 nm (black), close to the maximum at 598 nm of thionin acetate solution (red), attributed to H-type dimers of the dye and/or a vibrational band assigned to the n–π^* transitions of the C=N bond. Other feature that is worth to highlight is a great light scattering because of the nanomaterial suspension compared with the clear solution of thionine. As can be deduced from UV-Vis spectra, CDs have been effectively functionalized with thionin, obtaining a new material with important differences in the absorption spectra. Figure S1D shows Cyclic voltammograms (CV) of Ty-CDs, CDs and thionin acetate solution using a carbon screen-printed electrode (CSPE). In the CV of Ty-CDs, two reversible redox process at formal potential of -0.38 V and -0.54 V *vs.* Ag/AgCl are observed (black). Each of them exchanges one electron (obtained by Tafel plot). No redox process is observed at CDs CV (blue), which indicates that no redox process occurs. Thionin solution CV (red) shows a redox couple at a formal potential of -0.38 V, which involves two electrons. These results evidenced again the thionin modification of CDs, suggesting that the reaction is not a simple adsorption of the colorant over the CDs but rather that it has been embedded in the nanomaterial structure. XPS spectra of the N 1s and S 2p core level of Ty-CDs (black), CDs (blue) and thionin acetate (red) are reported at Figures S1E and S1F, respectively. All the spectra have been normalized in intensity to better observe the small changes in shape and chemical shifts. At N 1s peak, there is mainly one component in all samples, the chemical shift that the N 1s of Ty-CDs (black) suffer of 0.2 eV towards higher binding energies compare with CDs (blue).

Furthermore, in the S 2p core level, S is detected in the Ty-CDs (black) while it is not present in CDs (blue). Both results point towards a successful anchoring of the thionin on the CDs. In conclusion, we can confirm that Ty-CDs have been successfully generated, dispelling the doubt of just a physical adsorption of thionin over CDs surface.

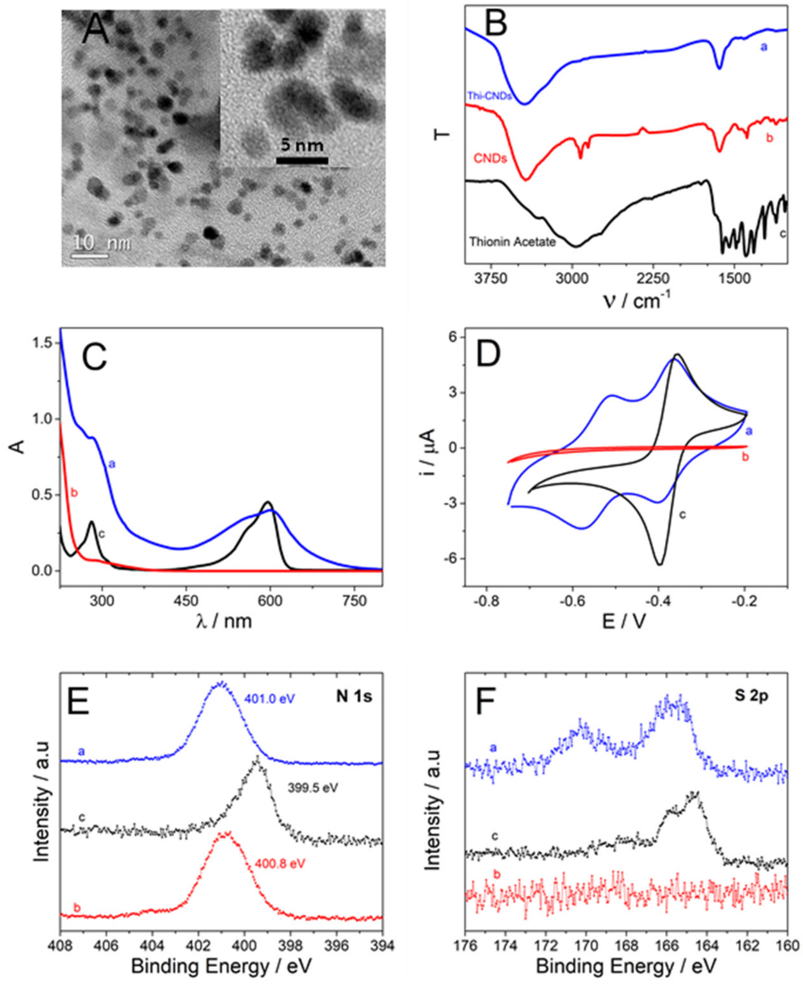

Figure S1. TEM micrographs of Ty-CDs (Inset: diameter histogram from TEM measurements of 120 Ty-CDs) (**A**), FT-IR of Ty-CDs, CDs and thionin acetate (**B**), UV-Vis spectra of Ty-CDs, CDs and thionin acetate (**C**), Cyclic voltammetry of Ty-CDs, CDs and thionin acetate in 0.1 M PB pH 7.0 buffer at 0.01 Vs^{-1} (**D**), XPS spectra for N 1s (**E**) and S 2p (**F**) core levels of Ty-CDs, CDs and thionin acetate.

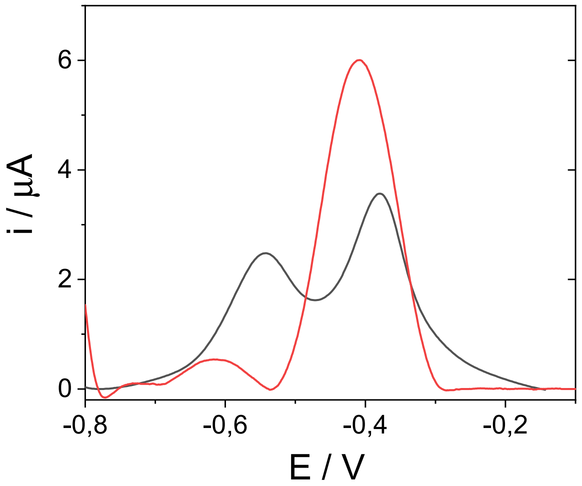

Figure S2. DPV of the biosensor signal in 0.1 M PB pH 7.0 buffer after incubation with 100 pM Listeria complementary sequence accumulating Ty-CDs by successive scans of potential (100 scans in a potential range of -0.70 to -0.15 V at 100 mV s^{-1}) (black curve) or via incubation at open circuit, adding 10 µL of 0.280 mg mL^{-1} Ty-CDs for 1 hour (red curve).

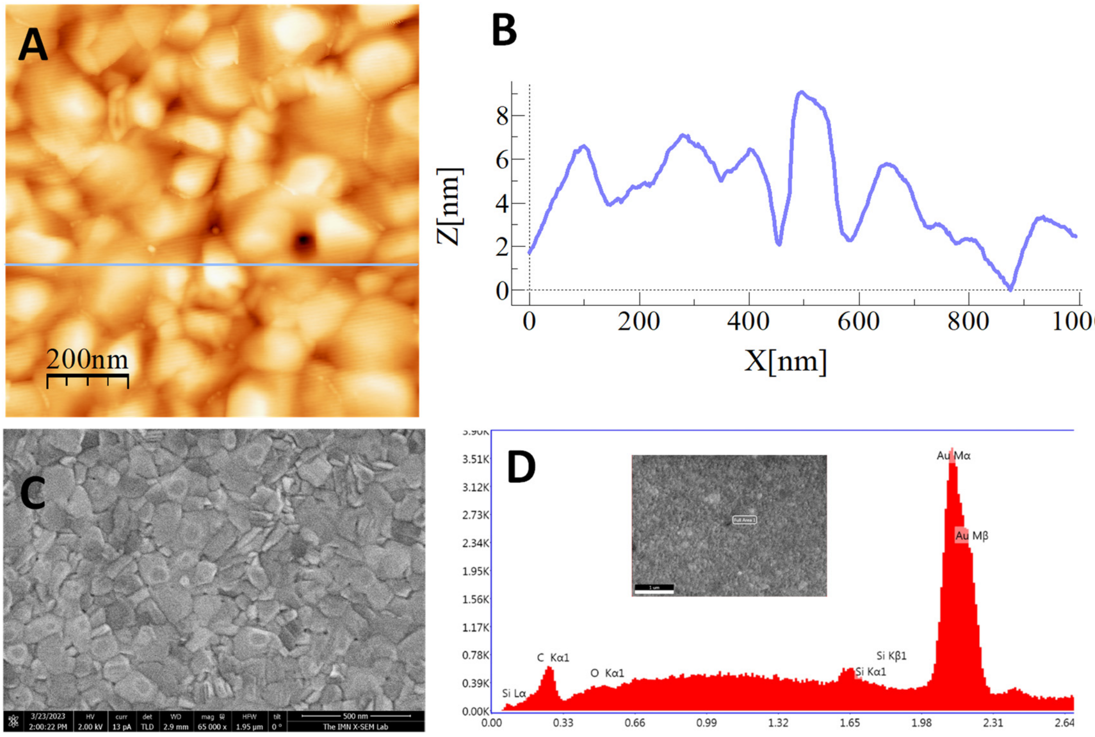

Figure S3. AFM image (**A**), a profile from it (**B**), SEM image (**C**) and EDAX spectra (**D**) of a bare gold substrate. Gold nanocrystals are observed in the images and the peak corresponding to the energy of gold can be clearly identified in the spectra.
